# Diagnostic effect of extended focussed assessment with sonography for trauma (E-FAST) in thoracic and abdomen injuries at a tertiary care centre

**DOI:** 10.6026/973206300223736

**Published:** 2026-06-30

**Authors:** Vrashabhan Ahirwar, Akhilesh Ratnakar, Ritesh Yadav, Talha Saad, Shreya Shrikant Chitode, Satyendra Mishra, Neelu Jain

**Affiliations:** 1 Department of Anaesthesiology, Bundelkhand Medical College Sagar, Madhya Pradesh, India; 2 Department of General Surgery, Bundelkhand Government Medical College, Sagar, Madhya Pradesh, India; 3 Department of General Medicine, SRVS Medical College, Shivpuri, Madhya Pradesh, India; 4 Department of Respiratory Medicine, Bundelkhand Medical College Sagar, Madhya Pradesh, India; 5 Department of Surgery, Bundelkhand Medical College, Sagar, Madhya Pradesh, India; 6 Department of Respiratory Medicine, Bundelkhand Medical College Sagar, Madhya Pradesh, India; 7 Department of Microbiology, Bundelkhand Government Medical College, Sagar, Madhya Pradesh, India

**Keywords:** Extended focussed assessment with sonography for trauma (E-FAST), blunt trauma, abdomen and chest

## Abstract

Extended focussed assessment with sonography for trauma (E-FAST) has become a paramount tool in emergency trauma department. Therefore, it is of interest to assess the effectiveness of E-FAST in individuals presenting with blunt abdominal and thoracic trauma, to evaluate its accuracy in guiding operative and conservative management strategies and to determine its role in improving triage efficiency, minimizing treatment delays and enhancing overall patient outcomes. Male individuals exhibited the greatest percentages of trauma than females and abdominal injuries were more frequent than thoracic injuries. The prominent mode of trauma was road traffic accidents with E-FAST examination being completed within 2-5 minutes in most of the cases. Thus, data shows the E-FAST is a prominent tool in ameliorating clinical judgment by expediting early recognition of fatal injuries and administering pertinent interventions.

## Background:

The chief determinant of fatal injuries and mortality around the globe is blunt trauma comprising of thoracic and abdomen region. The hour is condemning in care of injury. A minor setback in recognition of visceral injuries can remarkably hastens the likelihood of impediments and death. This necessitates the requisite of fast, reliable marker tool to be executed at the bedside of patient by emergency doctor. Extended focused assessment with sonography for trauma (E-FAST) has gained popularity as the most valuable instrument in the department of emergency medicine. E-FAST enables a swift and non-invasive approach to rule out the internal injuries leading the physicians for prompt intervention [1]. E-FAST is a type of ultrasonography enabling the swift examination at bedside and without the need of composite patient preparation and radiation exposure to the patients. The utilization of point-of-care ultrasound in the department of emergency has powered the early evaluation of injury [2, 3]. Previous studies have shown that E-FAST has increased diagnostic precision in disclosing blunt trauma to abdomen and chest, enabling the doctors to decide the requisite for operation and conservative treatment. The contrasting research between contrast enhanced CT and E-FAST have discovered that E-FAST is definitive for the detection of traumas leading to abdomen and chest region. These observations uncovered the gravity of its usage at bedside ultrasonography in emergency care because of its handy, quick, safety and usability [4, 5]. If the management got impeded it becomes grave in case of blunt trauma to thoracic and abdomen region. The chances of survival diminish with each passing minute when there is visceral bleeding enabling the quick action vital within the golden hour [6]. Therefore, it is of interest to evaluate the effectiveness of E-FAST in thoracic and abdomen injuries and its impact on treatment choices and its cumulative influence on better outcomes.

## Materials and Methods:

## Research design and sample:

This current prospective observational research comprised of 98 patients and was executed over a two-year period, from April 2023 to March 2025. All the subjects reporting to the emergency care setting with a history of traumas to the chest and abdomen because of high-risk mechanisms were evaluated suitability. Only patients who were hemodynamically stable after the initial evaluation were enrolled. The subjects who were younger than 18 years of age, pregnant and suffering only limb traumas or if imaging or during operatory observations were the exclusions. The subjects were excluded who failed to exemplify the post-training competency, if their E-FAST examination was executed by healthcare professionals. The subjects were excluded if they were not able to give informed consent. The sizes of the subjects were computed by implementing the aforementioned equation to proceed further with the research. Equation is N = z2 x p (1 - p ) / 2, wherein z refers the z score, N referring the size of population, p mentions the population proportion. Population portion was assumed to 50%, margin of error presumed is 10%, at 95% confidence interval and the size of the subjects came out to be 97. We preceded our research on 98 patients. The subjects who were enrolled for the research were scrutinized by emergency trauma centre professional educated in both basic and advanced ultrasound methods. The healthcare professional with no experience to ultrasonography had a structured 2-hour E-FAST training session executed by principal investigator, comprising of both theoretical and practical training session. This training approach was designed in accordance with established evidence indicating that such duration is sufficient to achieve fundamental competency in emergency ultrasound. The trainers had to undergo and interpret 10 E-FAST exams on injured cases before undergoing independent execution. A certified radiologist then reviewed these scans to confirm adequate interpretation skills. Competency was defined as correct interpretation of all 10 consecutive examinations.

## Statistical analysis:

By implementing the SPSS software, all the outcomes were computed and tabulated utilizing the descriptive statistics comprising of frequency and percentages.

## Results:

In the present research, time taken to perform the E-FAST procedure was 2-5 hours in greater than half of the population (56.1%) as seen in Table 1. RTA accounted to 57.1% of the patient's type of injury, followed by motor vehicle injury (25.5%) of the cases. When accounting for the region affected by injury to the thorax was the least found with 23.4% of the patients and the greatest was seen due to blunt injury to abdomen in 47.9% of the patients. 15.3% of the study population had HTN while 22.4% of them had both HTN and diabetic showing a good amount of patients with metabolic disorder.

## Discussion:

The usefulness of repeated E-FAST in patients suffering from blunt injuries to the chest and abdomen is reported in literature. Yazici *et al.* assessed the effectiveness of repeated E-FAST in stable trauma patients and highlighted its importance in the early recognition and continuous monitoring of internal injuries. These observations confirm that E-FAST is not only valuable during the initial assessment but is equally important in follow-up care to observe injury progression [7]. Attia *et al.* evaluated blunt thoracic injuries in polytrauma patients by comparing the National Emergency X-Radiography Utilization Study (NEXUS) chest protocol with e-FAST. Their results showed that E-FAST provided superior early detection of chest injuries [8]. This study emphasized that E-FAST is especially useful in emergency situations where rapid decision-making is essential. Its better performance than routine chest radiography in selected cases supports its use as a primary diagnostic tool for thoracic trauma. These findings further reinforce the growing evidence that E-FAST significantly improves early diagnosis in polytrauma patients with suspected chest injuries. The diagnostic accuracy of E-FAST has also been confirmed through systematic reviews and meta-analyses [9]. Netherton *et al.* reviewed multiple studies and concluded that E-FAST has high sensitivity and specificity in identifying thoracoabdominal injuries. Their analysis confirmed that E-FAST is a dependable imaging modality and often demonstrates better diagnostic performance compared to other investigations. These pooled data highlight the strong diagnostic capability of E-FAST and its important role in trauma care [10]. In the present study, blunt trauma was most commonly observed in young adults aged between 18 and 30 years. Similar trends were reported by Bella *et al.* [1] A clear male predominance was observed in our study, which is consistent with findings reported by Bella *et al.* and Clarke *et al.* [1, 6] Clarke *et al.* documented that 72.4% of patients undergoing E-FAST were male, closely matching another study where 74% of patients were men [6]. This male predominance may be attributed to the higher disability-adjusted life-years associated with transport-related injuries in males. Singh *et al.* and Pant *et al.* also reported a male preponderance in road traffic collisions. This trend is likely related to greater male involvement in outdoor occupations and high-risk activities such as driving, construction work and heavy physical labour [11, 12]. Most patients in our study had no significant pre-existing medical conditions, followed by those with hypertension and diabetes mellitus, findings that are in agreement with Bella *et al.* [1]. Abdominal trauma was the most frequent injury pattern, followed by combined abdominals-thoracic injuries, similar to the observations made by Bella *et al.* and Clarke *et al.* [1, 6] Road traffic accidents were the leading cause of injury in our study, followed by motor vehicle-related trauma, which is consistent with results reported in other studies. Payal *et al.* and Kithinji *et al.* identified falls and road traffic collisions as the most common mechanisms of injury, respectively [13, 14]. In the current study, the majority of E-FAST examinations were completed within 2-5 minutes, which aligns with the findings of Clarke *et al.*, who reported that 70.6% of examinations were performed within this time period [6]. Basnet *et al.* also observed that most E-FAST scans were completed in less than five minutes [3]. This study once again highlights the importance of E-FAST as an initial assessment tool in patients with blunt trauma. Its ability to provide quick, safe and dependable bedside examination makes it a valuable part of emergency trauma care. Future research should continue to explore wider applications of E-FAST and refine its diagnostic accuracy to further improve patient management and outcomes.

## Conclusion:

We show that E-FAST is an effective, quick, accurate and useful diagnostic tool for the early identification of thoracic and abdominal injuries in emergency trauma patients. It also aimed to evaluate how E-FAST can influence future management choices, such as whether more imaging, clinical monitoring, or surgery is required. Thus, the significance of repeat E-FAST assessments in patients with initially negative results to improve the diagnosis of emerging injuries is shown.

## Figures and Tables

**Table 1:** Descriptive analysis of variables of the current research

**Variables**		**N**	**Percentage**
Drug intake of the subjects	HTN	15	15.30%
having medical condition	HTN and diabetic	22	22.40%
	No significant history	61	62.20%
Regions affected by injury	Blunt Injury to abdomen	47	47.90%
	Blunt injury to thorax	23	23.40%
	Blunt injury to abdomen and thorax	28	28.50%
Type of injury	Fall and jump	3	3.06%
	Physical assault	6	6.10%
	RTA	56	57.10%
	Motor vehicle	25	25.50%
	Others	8	8.10%
Golden hour	Less than equal to 1 hour	29	29.50%
	Greater than 1 hour	69	70.40%
Time taken to perform	2-Jan	31	31.60%
e-FAST (in hours)	5-Feb	55	56.10%
	>5	12	12.20%
